# Use of pharmacotherapies for treatment resistant depression in finland: A nationwide cohort study

**DOI:** 10.1192/j.eurpsy.2021.311

**Published:** 2021-08-13

**Authors:** H. Taipale, M. Lähteenvuo, A. Tanskanen, S. Rannanpää, J. Tiihonen

**Affiliations:** 1 Forensic Psychiatry, Niuvanniemi Hospital, Kuopio, Finland; 2 Department Of Clinical Neuroscience, Karolinska Institutet, Stockholm, Sweden; 3 Janssen-cilag, Janssen-Cilag, Espoo, Finland

**Keywords:** Treatment Resistant Depression, pharmacotherapy

## Abstract

**Introduction:**

There is a lack of knowledge on utilized pharmacotherapies for treatment resistant depression (TRD).

**Objectives:**

To investigate the courses of treatment of TRD.

**Methods:**

All patients aged 16-65 years and diagnosed with depression in Finland during 2004-2016 were included (identified from nationwide registers for inpatient and specialized outpatient care, sick leaves and disability pensions). New antidepressant users were identified with six-month washout period and followed up for two years to observe the possible emergence of TRD, which was defined as initiation of a third treatment after having two failed pharmacological treatments with adequate duration. Pharmacological treatments were analyzed using PRE2DUP-method.

**Results:**

During follow-up, 177,144 persons had their first registered depression (mean age:39.5, 62.5% women). Of them, 10.9% (N=19,322) met TRD criteria. Among the TRD patients, most common first and second lines antidepressants were as follows: SSRIs (44.6%), mirtazapine (19.0%) and SNRIs (16.5%). As the third line of treatment, 44.2% of TRD patients had antidepressant monotherapy, 32.1% a combination of ≥2 antidepressants, 15.8% antipsychotic or mood stabilizer augmentation and an antidepressant, 4.9% both combination of antidepressants and an augmentation with a mood stabilizer or antipsychotic, 2.7% antipsychotic or mood stabilizer monotherapy and 0.3% ECT monotherapy. Of TRD patients, 36.2% (N=6985) progressed to the fourth line of treatment and most common treatments were antidepressant monotherapy (37.5%), antidepressant combinations (30.8%) and augmentation (20.3%).

**Conclusions:**

Although antidepressant combination and augmentation strategies became more frequent, antidepressant monotherapies were still the most common third and fourth lines of depression treatment.

**Disclosure:**

The study was funded by Janssen and SR is an employee of Janssen.

